# Fibroblast activation protein in the tumor microenvironment predicts outcomes of PD-1 blockade therapy in advanced non-small cell lung cancer

**DOI:** 10.1007/s00432-022-04250-4

**Published:** 2022-08-11

**Authors:** Yan Zhao, Yueping Liu, Yunlong Jia, Xiaoxiao Wang, Jiankun He, Shuman Zhen, Jiali Wang, Lihua Liu

**Affiliations:** 1 Department of Tumor Immunotherapy, The Fourth Hospital of Hebei Medical University, Shijiazhuang, 050035 China; 2 Department of Oncology, The Fourth Hospital of Hebei Medical University, Shijiazhuang, 050011 China; 3 Department of Pathology, The Fourth Hospital of Hebei Medical University, Shijiazhuang, 050011 China; 4 Cancer Research Institute of Hebei Province, Shijiazhuang, 050011 China; 5 China International Cooperation Laboratory of Stem Cell Research, Hebei Medical University, Shijiazhuang, 050011 China

**Keywords:** Fibroblast activation protein, Programmed cell death-1 blockade, Cancer-associated fibroblasts, Tumor microenvironment, Non-small cell lung cancer, Biomarker

## Abstract

**Purpose:**

The identification of robust predictive biomarkers of the response to programmed cell death-1 (PD-1) blockade remains a critical concern. Here, we investigated on fibroblast activation protein (FAP) as a microenvironment-derived biomarker of clinical outcomes of PD-1 blockade therapy, and the correlation between FAP expression and T cell infiltration in advanced non-small cell lung cancer (NSCLC).

**Methods:**

A total of 135 patients with advanced NSCLC who received PD-1 blockade therapy were retrospectively analyzed. The potential associations among FAP expression, CD3 + T cell and CD8 + T cell infiltration, and clinical outcomes of immunotherapy were validated by immunohistochemistry, bioinformatic analyses, and statistical measurements.

**Results:**

FAP was widely expressed in advanced NSCLC tissues. FAP was correlated with decreased density of CD8 + T cells (Spearman’s rho  – 0.32, *p* < 0.001) and immunosuppressive tumor microenvironment (TME) status. No correlations were detected between FAP and PD-L1 expression or with the density of CD3 + T cells. The patients with higher expression of FAP showed worse response rate (16.4% *vs.* 38.7%, *p* < 0.001) and worse progression-free survival (HR = 2.56, 95% CI 1.69–3.87, *p* < 0.001). In addition, FAP contributed to shortened overall survival in subgroups of the patients with squamous cell lung cancer (*p* = 0.020), PD-1 blockade monotherapy (*p* = 0.017), and first-line therapy (*p* = 0.028).

**Conclusion:**

FAP is a potential predictive biomarker of resistance to PD-1 blockade. Further investigation is warranted to identify a strategy for targeting FAP to alleviate the immunosuppressive TME and broaden the clinical effectiveness of PD-1 blockade therapy.

**Supplementary Information:**

The online version contains supplementary material available at 10.1007/s00432-022-04250-4.

## Introduction

Immune checkpoint blockade (ICB) therapy has shifted the paradigms of cancer management and become the cornerstone of systematic therapy of advanced NSCLC (Reck et al. 2022; Carbone et al. 2017; Gandhi et al. 2018). Among the ICB strategies, programmed cell death-1 (PD-1) blockade is the most widely applied therapy worldwide. However, the response rates of therapy with PD-1 blockade among advanced NSCLC patients are highly heterogeneous, ranging from approximately 10–50% (Hirsch et al. 2019; Garon et al. 2015), leading to an unmet need for improving clinical outcomes. As the most widely applied companion biomarker for PD-1 blockade therapy in NSCLC, detection of PD-L1 on tumor cells is of limited clinical value due to its unsatisfactory predictive power, varying thresholds, as well as spatial and temporal variations (Mansfield et al. 2016; Ilie et al. 2016). These clinical findings highlight ongoing searches for robust biomarkers that could preselect patients who would derive the maximum benefit from PD-1 blockade therapy.

The tumor microenvironment (TME) is a dynamic system where tumors initiate, grow, and metastasize; therefore, the status of the TME usually determines the outcomes of cancer patients (Petitprez et al. 2020). The TME consists of tumor cells, immune cells, vascular endothelial cells, and stroma cells (Joyce and Fearon 2015). Emerging studies have revealed that complex variations of the TME are critical in the process of cancer progression and cancer immunotherapy. Cancer-associated fibroblasts (CAFs) are the predominant stromal cell type, and they actively participate in remodeling the immunosuppressive microenvironment in NSCLC. Bugaev and colleagues identified four distinct types of TME as follows: immune-enriched, fibrotic; immune-enriched, nonfibrotic; fibrotic; and immune-depleted (Bagaev et al. 2021). The abundance and activation of CAFs have become indispensable markers in this classification and are associated with the worst prognoses of the patients receiving ICB therapy. In addition, low response rates upon PD-1 blockade therapy are attributed to the paucity of pre-existing tumor infiltrating lymphocytes (TILs), of which CD3 + T cells (total T lymphocytes) and CD8 + T cells (T effector cells) act as major mediators of the adaptive immune response (Galon et al. 2006; Waldman et al. 2020). Evidently, CAFs are correlated with a decreased abundance of TILs and poor outcomes of immunotherapy (Kieffer et al. 2020; Dominguez et al. 2020; Bagaev et al. 2021). Of note, CAFs exhibit phenotypic heterogeneity, and fibroblast subtypes have been established in breast cancers, pancreatic cancers and NSCLC, defining distinct functions in the TME (Ogawa et al. 2021; Costa et al. 2018; Bouchard et al. 2022; Hu et al. 2021; Galbo et al. 2021). However, the existing CAFs subgroups still fail to meet the clinical demands as predictive biomarkers of response to ICB (Wang et al. 2021), and the impacts of diverse CAF-related molecules on the immunotherapy response and their correlation to T cells remain to be elucidated.

One molecule of particular interest on the CAF is fibroblast activation protein (FAP), which has been discovered to be widely expressed in the pan-cancer stroma. FAP is a representative marker of activated CAFs and elevated expression of FAP promotes tumor progression by regulating multiple biological processes related to tumor cell growth, invasion, metastasis, drug resistance and stemness, and is associated with a worse prognosis across most cancer types (Fitzgerald and Weiner 2020). Moreover, FAP-positive CAFs contribute to the remodeling of the immune contexture in the TME by secreting cytokines and chemokines (Feig et al. 2013), enhancing the recruitment of immunosuppressive cells (Yang et al. 2016), and upregulating immune checkpoint molecules (Érsek et al. 2020), thereby dampening the immunotherapeutic response. Targeting stromal cells expressing FAP has been proven to improve the immune response and results in increased sensitivity to PD-1 blockade in mouse models (Kraman et al. 2010; Chen et al. 2017; Wen et al. 2017). Moreover, FAP contributes to primary resistance to immunotherapy in metastatic urothelial cancer and breast cancer patients (Powles et al. 2019; Kieffer et al. 2020). Notably, although FAP expression was reported as an independent biomarker of a poor prognosis for lung adenocarcinoma (LUAD) (Moreno-Ruiz et al. 2021), none of the previous studies have confirmed the possibility that FAP is linked to the outcome of PD-1 blockade therapy and immune infiltrates in advanced NSCLC. We hypothesized that the expression of FAP in advanced NSCLC patients might serve as a surrogate marker that can stratify PD-1 blockade-treated patients into groups that benefit from blockade treatment and those that do not. This concept needs to be validated in clinical settings for NSCLC through integrated analyses of CAF markers and T cell infiltration.

In this study, we explored the expression of FAP in the tumor stroma of advanced NSCLC tissues and determined whether high expression of FAP was associated with decreased immune infiltrates, particularly on CD3 + T cells and CD8 + T cells in the TME. Moreover, we evaluated the clinical value of FAP for predicting the immunotherapeutic outcomes in a cohort of patients with advanced NSCLC who received PD-1 blockade therapy, which yielded a more complete understanding of interactions between FAP and T cell infiltration and uncovered their roles in the immunotherapeutic response.

## Materials and methods

### Patients and clinical samples

The study cohort was retrospectively selected from advanced NSCLC patients who received PD-1 blockade at the Fourth Hospital of Hebei Medical University from January 2015 to November 2020. Key criteria of enrollment included unresectable, metastatic or recurrent stage IIIB–IV NSCLC, according to the 8th edition of the American Joint Committee on Cancer (AJCC) TNM staging classification (Wankhede 2021), pathologically confirmed diagnosis, provision of tumor samples from surgery, percutaneous lung biopsy or bronchoscopy biopsy before therapy, an Eastern Cooperative Oncology Group performance status (ECOG PS) of 0–2, at least one measurable lesion according to the Response Evaluation Criteria in Solid Tumors (RECIST) version 1.1 (Eisenhauer et al. 2009), and adequate organ function. Patients with a history of systemic immunosuppressive therapy, noninfectious pneumonitis, or active autoimmune disease requiring steroid therapy were excluded. Clinical information was collected from the hospital database, which was updated every 3 months by clinical or telephone follow-up.

### Immunohistochemistry (IHC)

IHC staining was performed according to previously described protocols (Renga et al. 2022). Briefly, slides were baked in an oven at 65 °C, deparaffinized, dehydrated and subjected to heat-induced antigen retrieval with EDTA (pH 9.0) for FAP and CD8α and with citrate buffer (pH 6.0) for CD3. The sections were incubated overnight with primary antibodies against FAP (rabbit, 1:250, ab207178; Abcam, USA), CD3 (rabbit, 1:150, ab16669; Abcam) and CD8α (rabbit, 1:250, ab217344; Abcam) at 4 °C. After washing with PBS, the sections were incubated with anti-rabbit HRP for 30 min at room temperature and then treated with diaminobenzidine. The sections were then counterstained with hematoxylin, dehydrated with an ethanol series, cleared in xylene, mounted, and observed under a microscope.

### Scoring of IHC

To evaluate the expression of FAP in NSCLC tumor tissues, the IHC staining was scored by a semiquantitative method based on the intensity of FAP staining and the percentage of FAP-positive cells in the tumor stroma compartment (Moreno-Ruiz et al. 2021). FAP intensity of the positively stained stroma was graded as 0: no staining; 1 + : low intensity; 2 + : medium intensity; 3 + : high intensity. The percentage of FAP-positive cells was scored as follows: grade 0 (absent or < 1%), grade 1 (1–10%), grade 2 (11–50%), grade 3 (51–75%), and grade 4 (76–100%). A composite IHC score was determined by multiplying the staining intensity score and the percentage of positive cells (Sideras et al. 2017). The cut-off threshold set at a median value of 6 was applied for dichotomization of FAP; a score of > 6 was considered high FAP expression, and ≤ 6 was considered low FAP expression.

The percentages of CD3- and CD8-positive lymphocytes compared with the entirety of the nucleated cells were manually scored based on cell counting in ten randomly selected high-power fields of tumor cores. The resulting patient-based scoring was the average percentage of the core-based scoring, which was then categorized into low- density and high-density groups using the median value of the core score as a cut-off value (10% for CD3 and 5% for CD8): low density of CD3 + T cells: ≤ 10%; high density of CD3 + T cells: > 10%; low density of CD8 + T cells: ≤ 5%; high density of CD8 + T cells: > 5% (Dong et al. 2017).

PD-L1 status was assessed with a commercial IHC kit by anti-22C3 antibodies or clone SP142 (Herbst et al. 2016), using established scoring criteria: tumor proportion score (TPS) and immune cell (IC) score. PD-L1 TPS expression was scored positive for analysis as > 1%, and PD-L1 IC expression was scored positive for analysis as ≥ 1%. The stained tissue sections were evaluated by two independent pathologists, and all scoring was performed blinded to the clinical parameters and outcome data.

### Clinical outcome evaluations

The clinical outcomes of this study included the objective response rate (ORR), disease control rate (DCR), progression-free survival (PFS), and overall survival (OS). Patients underwent tumor assessments at baseline, every 6–8 weeks for 48 weeks, and every 9–12 weeks thereafter until disease progression or treatment discontinuation. The best overall response was determined through RECIST V1.1 as complete response (CR), partial response (PR), stable disease (SD), or progressive disease (PD) (Eisenhauer et al. 2009). Patients who achieved CR/PR were defined as responders, and patients who achieved SD/PD were termed non-responders. PFS was calculated from the date of first immunotherapy administration until disease progression or death. OS was defined as the time of diagnosis until cancer-related death or the date of the last follow-up. Complete follow-up was performed on Mar 1, 2022.

### Additional bioinformatic analysis

Gene expression data of LUAD patients (*n* = 500) and squamous cell lung carcinoma patients (LUSC, *n* = 491) were obtained from The Cancer Genome Atlas (TCGA) database (https://xenabrowser.net/). Single-cell type information of FAP in NSCLC was acquired from the Tumor Immune Single Cell Hub (TISCH, http://tisch.comp-genomics.org) database (Sun et al. 2021). Immune and stromal scores were calculated based on the ESTIMATE algorithm (Yoshihara et al. 2013). An annotated gene set (c5.go.bp.v7.4.symbols.gmt) from the molecular signatures database was selected for gene set enrichment analysis (GSEA) (Subramanian et al. 2005). We also employed the Tumor Immune Estimation Resource database (TIMER, https://cistrome.shinyapps.io/timer/) and the Tumor Immune Dysfunction and Exclusion (TIDE) framework (http://tide.dfci.harvard.edu) to explore the association between FAP expression and the degree of immune infiltrates (Li et al. 2020; Jiang et al. 2018).

### Statistical analysis

Statistical analyses were performed using RStudio V3.6.1 (http://www.R-project.org/) and SPSS 26.0. The associations between FAP expression and the clinicopathological characteristics were evaluated by the chi-square test and Fisher’s exact test. The correlation of FAP and PD-L1 expression with the density of CD3 + T cells and CD8 + T cells was estimated by the Spearman rank correlation test. The potential prognostic factors in relation to PFS and OS were screened using log-rank tests, with results reported through Kaplan‒Meier graphs. The prognostic significance of the different parameters was analyzed using univariate or multivariate Cox proportional hazards models. All statistical tests were two-tailed, and *p* values < 0.05 were considered statistically significant.

## Results

### Patient characteristics

Overall, a total of 135 patients who received PD-1 blockade therapy were included in this study. The details of the patients are summarized in Table 1. Among these clinicopathological characteristics, the median age was 63 years (range: 29–82) at diagnosis. Most patients were men (80.0%), current or former smokers (57.8%), had tumors of non-squamous NSCLC histology (57.8%), and had an ECOG PS of 0–1 (87.4%). The whole cohort was consisted of 60 (44.4%) patients who received PD-1 blockade monotherapy and 75 (55.6%) patients who received PD-1 blockade therapy plus platinum-based chemotherapy. Sixty-five (48.1%) patients received PD-1 blockade therapy as first-line treatment. PD-L1 status was available for 73 (54.1%) patients and was determined as positive (≥ 1% of tumor cells) in 61 (83.6%) of these patients. At the cut-off date of Mar 1, 2022, the median follow-up time was 23.4 months (range, 2.2–86.2 months). The population included 78 (57.8%) deceased patients; among them, there was a remarkably higher proportion of patients with stage IV disease (85.9% *vs.* 57.9%, *p* < 0.001) and multiple distant metastases (42.3% *vs.* 31.6%, *p* = 0.004).

**Table 1:** Clinicopathological parameters of advanced NSCLC patients and correlation with FAP expression (total *n* = 135)

Clinicopathological parameter	Stratification	Frequency (proportion)	FAP^high^	FAP^low^	*p*-value
Age at diagnosis	< 60	49 (36.3%)	27	22	0.856
	≥ 60	86 (63.7%)	46	40	
Gender	Male	108 (80.0%)	59	49	0.796
	Female	27 (20.0%)	14	13	
Smoking history	Yes	78 (57.8%)	46	32	0.181
	No	57 (42.2%)	27	30	
Histological type	Non-squamous cell carcinoma	78 (57.8%)	47	31	0.092
	Squamous cell carcinoma	57 (42.2%)	26	31	
TNM Stage	IIIB	35 (25.9%)	14	21	0.052
	IV	100 (74.1%)	59	41	
LNM	Yes	98 (72.6%)	53	45	0.998
	No	37 (27.4%)	20	17	
Distant metastasis	Multiple	51 (37.8%)	39	12	< 0.001
	Solitary	43 (31.9%)	16	27	
	None	41 (30.3%)	18	23	
ECOG PS	≤ 1	118 (87.4%)	64	54	0.920
	2	17 (12.6%)	9	8	
Type of immunotherapy	Anti-PD-1 monotherapy	60 (44.4%)	36	24	0.217
	Anti-PD-1 + chemotherapy	75 (55.6%)	37	38	
Line of systematic treatment	First line	65 (48.1%)	37	28	0.804
	Second line	48 (35.6%)	25	23	
	Third line or later	22 (16.3%)	11	11	
Stroma fraction	≤ 50%	72 (53.3%)	41	31	0.474
	> 50%	63 (46.7%)	32	31	
CD3	High	53 (39.3%)	26	27	0.347
	Low	82 (60.7%)	47	35	
CD8	High	50 (37.0%)	23	27	0.149
	Low	85 (63.0%)	50	35	
PD-L1 (TPS) status	Positive	61 (45.2%)	32	29	0.654
	Negative	12 (8.9%)	8	4	
	NA	62 (45.9%)	33	29	
PD-L1 (IC) status	Positive	38 (28.2%)	26	12	0.051
	Negative	35 (25.9%)	14	21	
	NA	62 (45.9%)	33	29	

*LNM* lymph node metastasis

*ECOG PS* Eastern Cooperative Oncology Group Performance Status

*PD-1* programmed cell death-1

*PD-L1* programmed cell death ligand-1

*NA* not available

### Expression of FAP in the tumor stroma and associations with clinicopathological characteristics of advanced NSCLC

**Fig. 1:**
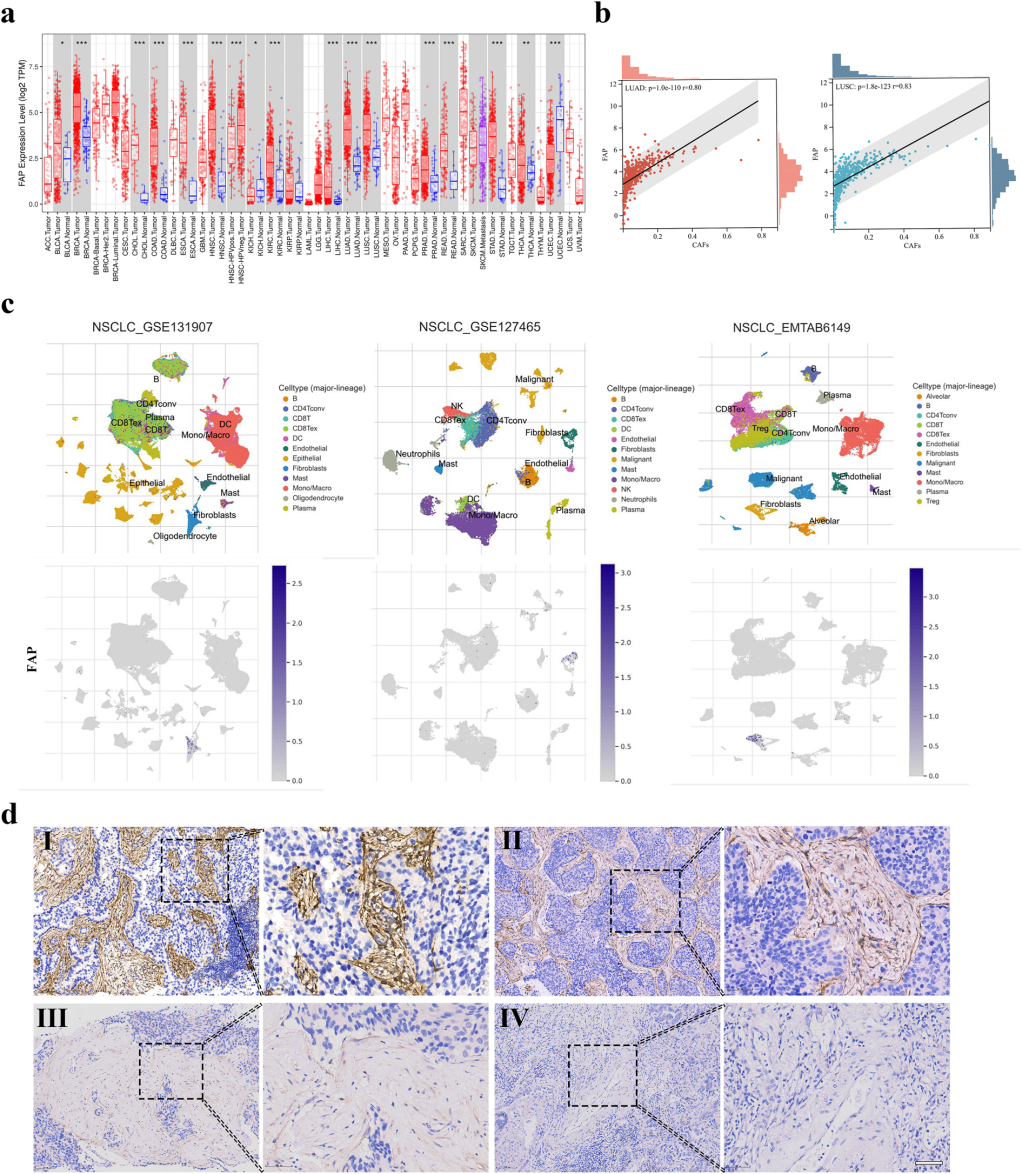
FAP expression in the stroma of advanced NSCLC tissues. **a** Expression of FAP in tumor tissues and normal tissues of the NSCLC cohort in the TCGA database. **b** Correlations of FAP with the cluster of CAFs in the TME of NSCLC. **c** FAP expression in NSCLC as visualized by single-cell analysis from the TISCH database. **d** Representative IHC images of tumors showing FAP staining: (I) high intensity, (II) medium intensity, (III) low intensity and (IV) negative staining. (brown = FAP; blue = hematoxylin nuclear staining). Scale bar = 50 µm. * *P* < 0.05; ** *P* < 0.01 and *** *P* < 0.001

To characterize the expression of FAP in cancers, we first analyzed the expression of FAP in tumor tissues and normal tissues from the TCGA database. FAP was upregulated in various types of cancers, including NSCLC (Fig. 1a), and showed a strong association with the cluster of fibroblasts (Fig. 1b, c). IHC analysis of advanced NSCLC tumor tissues showed that FAP was mainly expressed in the cytoplasm of CAFs in the tumor stroma, which was consistent with previous studies (Fitzgerald and Weiner 2020). Selected cases with different levels of FAP intensity are shown in Fig. 1d, which were defined as high, medium, low and negative staining. FAP expression covered 0–90% of the stroma surface area (median 60%). Among the 135 cases involved in this study, 73 (54.1%) were defined as having high FAP expression, and the remaining 62 (45.9%) cases were considered to have low FAP expression. Subsequent analysis was conducted to reveal the associations between FAP expression and clinicopathological characteristics. As shown in Table 1, FAP expression positively correlated with the presence of distant metastasis (*p* < 0.001). In addition, there were more patients with multiple distant metastases in the high FAP expression group than in the low FAP expression group (53.4% *vs.* 19.4%, *p* < 0.001). Nevertheless, FAP expression was not associated with age, gender, smoking history, histology, TNM stage, ECOG PS, or stroma fraction.

### Internal associations between FAP and PD-L1 expression and the density of T lymphocytes in the TME

**Fig. 2:**
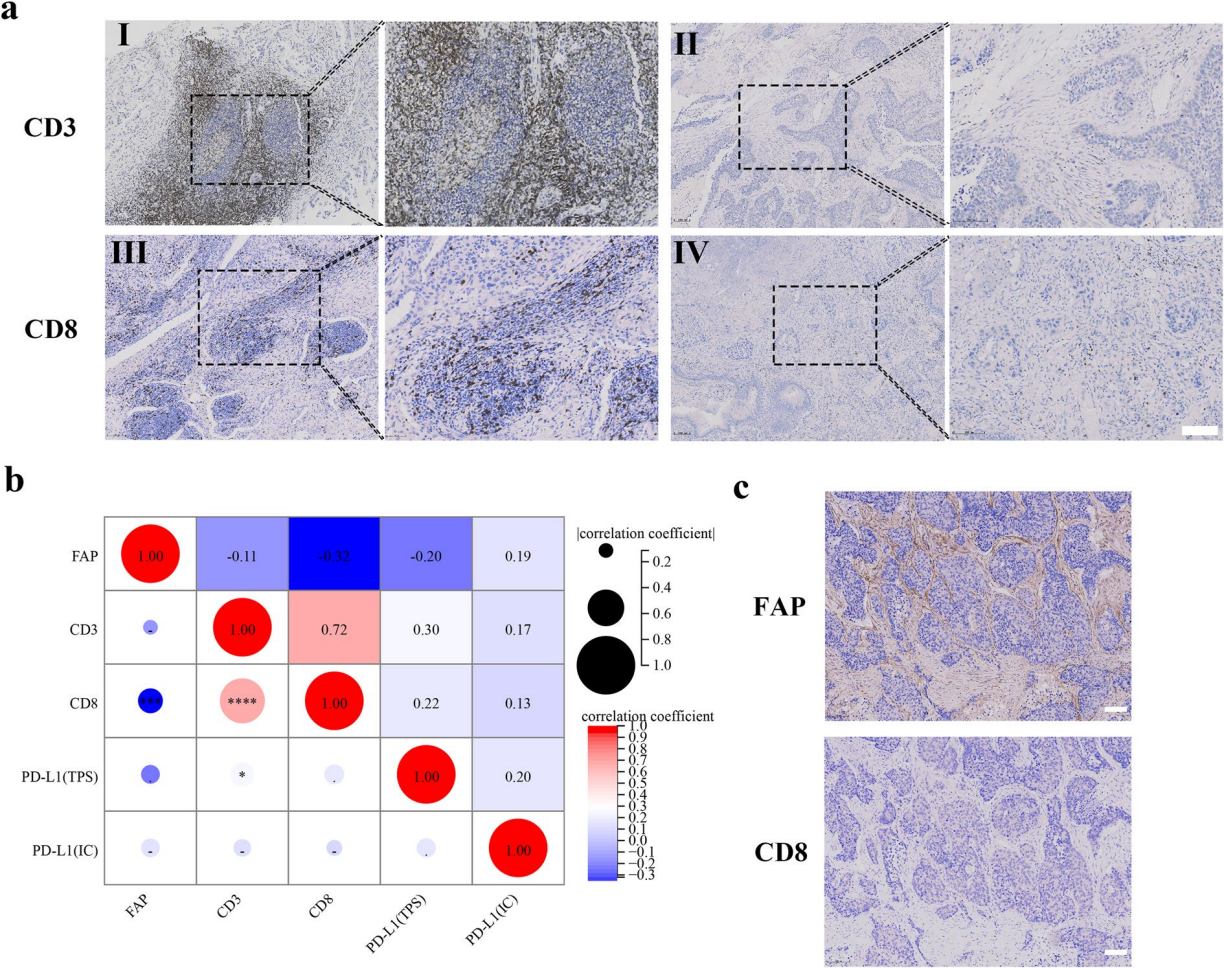
T-cell infiltration of advanced NSCLC tissues and intracase correlation between FAP and PD-L1. **a** Representative IHC images of tumors showing high (I) and low (II) density of CD3 + T cells; High (III) and low (IV) density of CD8 + T cells (brown = CD3 or CD8; blue = hematoxylin nuclear staining). Scale bar = 100 µm. **b** Correlations between FAP, T lymphocytes and PD-L1 in NSCLC patients. The figure shows the results of the Spearman two-tailed test. **c** Representative IHC images of NSCLC tissue showing inverse expression patterns of FAP and CD8. Scale bar = 100 µm. *P < 0.05; ***P < 0.001; ****P < 0.0001

As FAP has been reported to be involved in the remodeling of the TME, we next examined whether FAP expression was correlated with tumor-infiltrating T lymphocytes in NSCLC tissue. Representative IHC staining of CD3 + T cells and CD8 + T cells are shown in Fig. 2a. The percentages of CD3 + T cells and CD8 + T cells ranged from 0 to 60% (mean value 17%) and from 0 to 40% (mean value 7%), respectively. High FAP expression was negatively correlated with the density of CD8 + T cells (Spearman’s rho  – 0.32, *p* < 0.001, Fig. 2b, c), but not related to the density of CD3 + T cells.

Exploratory analysis in advanced NSCLC subsets related to histology indicated some differences in the strengths of these correlations. A particularly strong correlation was found in squamous cell lung cancer between FAP and CD8 + T cell density, which was stronger than in the non-squamous cell lung cancer group (Spearman’s rho  – 0.34, *p* = 0.01; Spearman’s rho  – 0.30, *p* = 0.008, respectively). Representative IHC staining of PD-L1 was shown in Supplementary Fig. 1. PD-L1 expression (TPS and IC score) did not show significant correlations with FAP or CD8 + T cell density, but PD-L1 expression of TPS was correlated with CD3 + T cell density (Spearman’s rho 0.30, *p* = 0.011) when analyzed in the whole cohort (Fig. 2b).

### FAP is associated with the immunosuppressive TME status in NSCLC

**Fig. 3:**
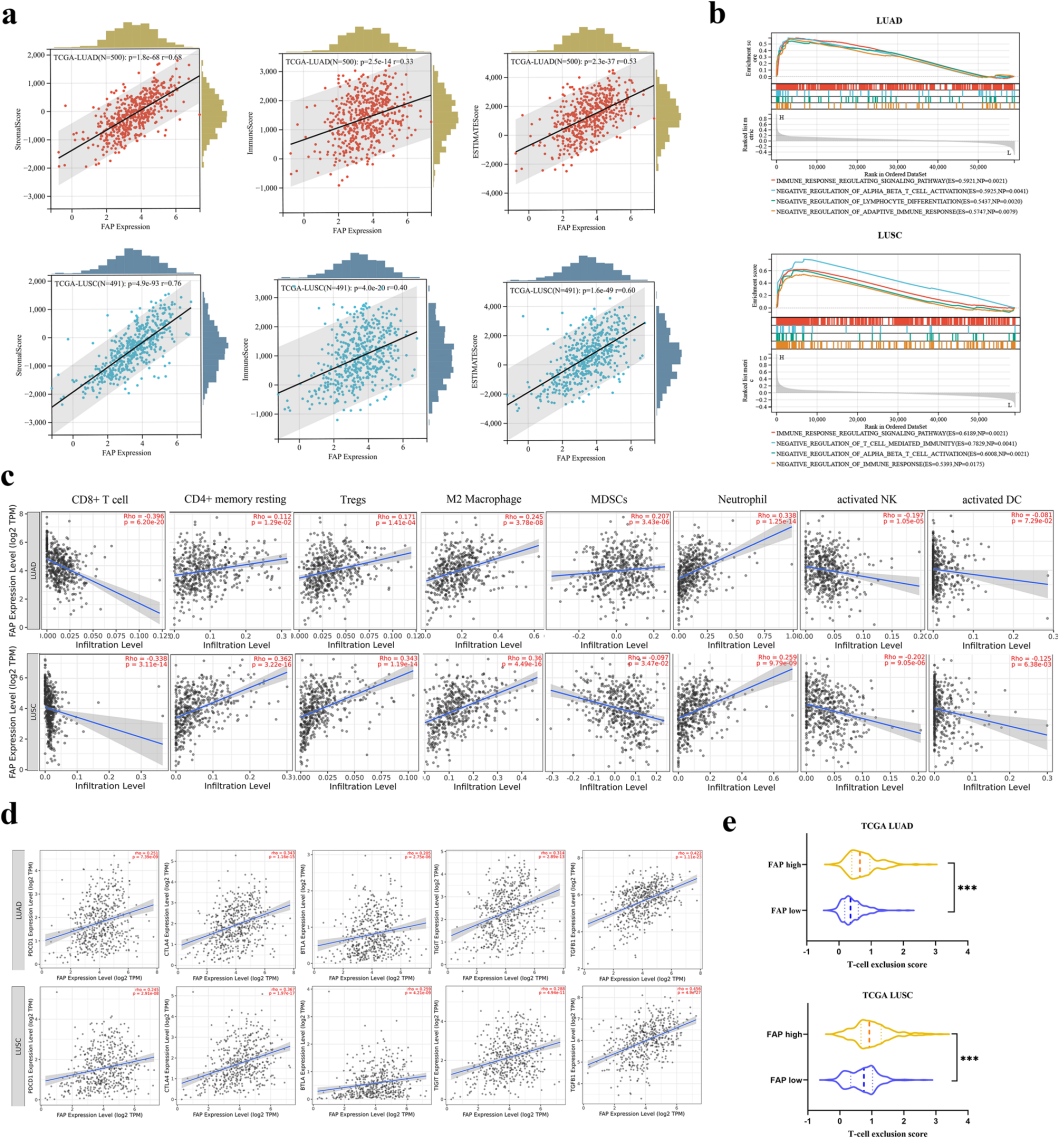
Correlation of FAP expression with immunosuppressive features in the TME of NSCLC. **a** Correlation of FAP expression between immune/stromal/ESTIMATE scores. **b** GSEA of FAP in LUAD and LUSC from the TCGA dataset. **c** Correlations between FAP and infiltrating levels of CD8 + T cells, CD4 + memory resting cells, Tregs, M2 macrophages, MDSCs, neutrophils, activated NK cells and activated DCs of LUAD and LUSC in the TIMER database. **d** Correlation of FAP expression with immune checkpoints and immunosuppressive genes in the TME of LUAD and LUSC in the TIMER database. **e** T cell exclusion score in the high- and low-FAP groups of LUAD and LUSC estimated by the TIDE framework. ***P < 0.001

To explore a more comprehensive role of FAP in the TME of NSCLC, we investigated the potential correlation of FAP and the immune and stromal scores in the TCGA cohort, which were processed by the ESTIMATE algorithm. We observed that FAP was significantly correlated with stromal scores (LUAD: *r* = 0.68; LUSC: *r* = 0.76, both *p* < 0.001), immune scores (LUAD: *r* = 0.33; LUSC: *r* = 0.40, both *p* < 0.001) and ESTIMATE scores (LUAD: *r* = 0.53; LUSC: *r* = 0.60, both *p* < 0.001), indicating that FAP was a TME-related gene (Fig. 3a). We next evaluated the immunological pathways in which FAP might be involved, by analyzing the NSCLC cohort in the TCGA database with GSEA. The results showed that the negative regulation of immune response pathways was significantly enriched in LUAD and LUSC patients with high FAP expression, especially for T cell-mediated immunoregulatory pathways (Fig. 3b). These results suggested that FAP was negatively correlated with the tumor immune response.

To elucidate the potential relationship between FAP expression and immune cell infiltration, we studied the features of FAP-correlated immune infiltration and immune-related genes using the TIMER database. The results showed that FAP expression was inversely correlated with the infiltration of CD8 + T cells, activated DCs and activated NK cells in both LUAD and LUSC patients (*p* < 0.01, Fig. 3c). Moreover, FAP expression was positively correlated with the abundance of immunosuppressive cells, such as Tregs, M2 macrophages, and neutrophils (all *p* < 0.001, Fig. 3c). FAP was also positively correlated with MDSCs in LUAD and CD4 + memory resting cells in LUSC (both *p* < 0.0001, Fig. 3c). Additionally, FAP was positively related to the upregulation of immune checkpoints and immunosuppressive genes, including PD-1, T cell protein cytotoxic T lymphocyte antigen 4 (CTLA4), T cell immunoglobulin and ITIM domain (TIGIT), B and T lymphocyte attenuator (BTLA) and transforming growth factor-beta (TGF-β) (all *p* < 0.0001, Fig. 3d). Moreover, the TIDE framework (Jiang et al. 2018) was applied to the analysis, indicating that patients in the high-FAP group were characterized by significantly higher T cell exclusion scores (*p* < 0.001, Fig. 3e). Taken together, these findings were consistent with the hypothesis that FAP was relevant to the features of an immunosuppressive TME in NSCLC, which highlighted the importance of stroma-based strategies in immunotherapy.

### Predictive value of FAP expression on immunotherapy response and tumor-specific survival for advanced NSCLC patients

**Fig. 4:**
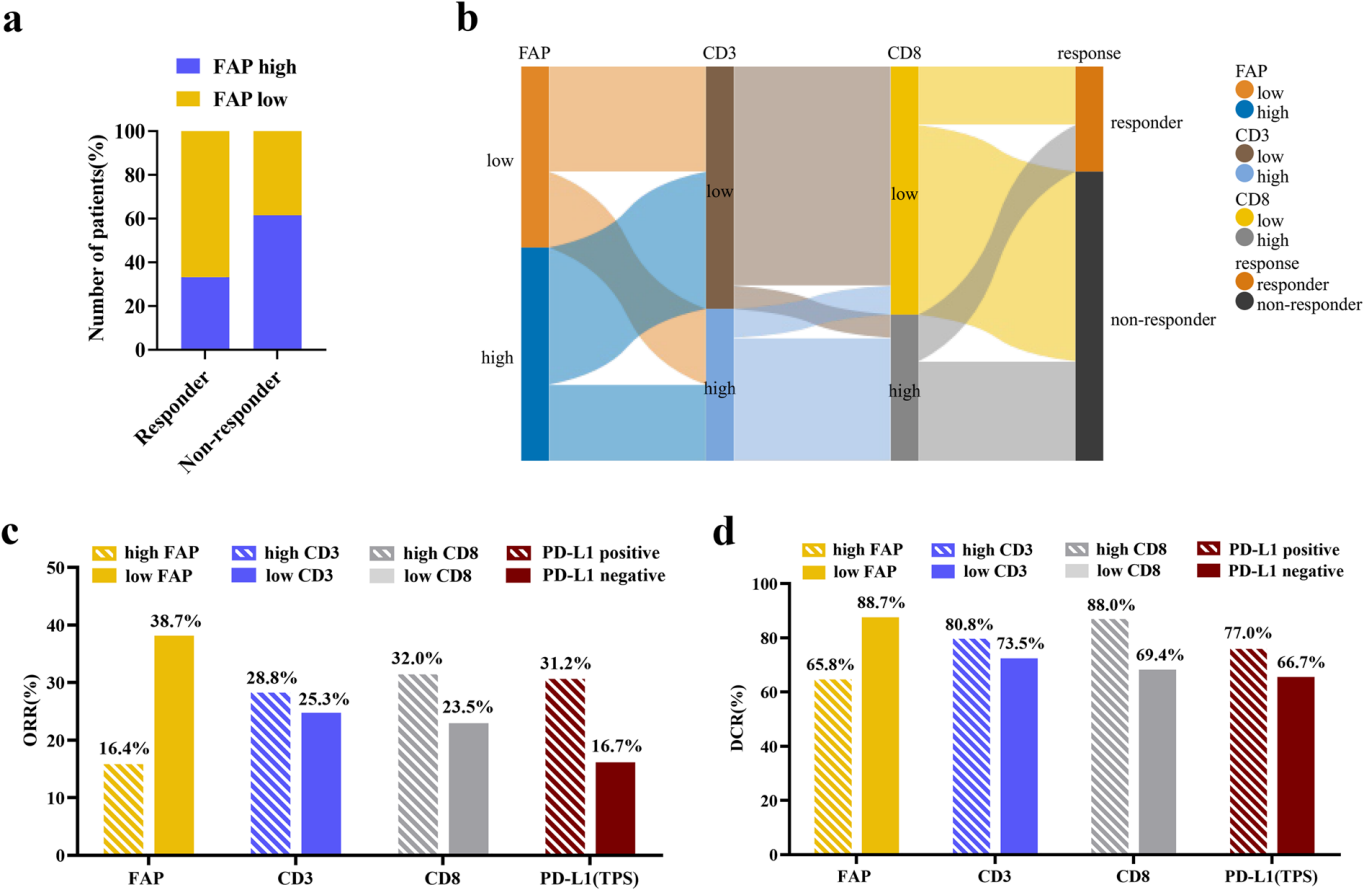
Response to PD-1 blockade in patients with advanced NSCLC in relation to FAP, T lymphocytes and PD-L1. **a** The proportion of patients with high/low FAP expression of responders and non-responders treated with PD-1 blockade. **b** Sankey diagram showing FAP expression in the low and high groups linked to different infiltration levels of T cells, and immunotherapeutic responses. **c** ORR for the predictive value of FAP, CD3 + T cells, CD8 + T cells and PD-L1 (TPS). **d** DCR for the predictive value of FAP, CD3 + T cells, CD8 + T cells and PD-L1 (TPS)

Next, we aimed to determine whether FAP expression had predictive value for the PD-1 blockade response. A significant difference in the level of FAP expression was observed between responders and non-responders to PD-1 blockade therapy. More patients with low-FAP expression were found in the responders’ group (*p* = 0.004, Fig. 4a). Sankey plots suggested that the patients with high FAP expression were mostly concentrated in the low density of CD3 + T cell group and low density of CD8 + T cell group, while these groups were preferentially trended towards the non-responders (Fig. 4b). Moreover, we compared the efficacy of PD-1 blockade according to different stratifications of FAP expression, the density of CD3 + T cells, the density of CD8 + T cells and PD-L1 expression. The ORR of the low FAP expression group was the highest, at 38.7%, while, the high FAP expression group conferred the lowest ORR, at 16.4% (Fig. 4c). Similarly, patients harboring low expression of FAP experienced a significantly better DCR of 88.7%, compared with 65.8% in patients with high FAP expression (Fig. 4d). These results underlined the possibility of FAP as a biomarker for predicting the efficacy of PD-1 blockade therapy in advanced NSCLC patients.

**Fig. 5:**
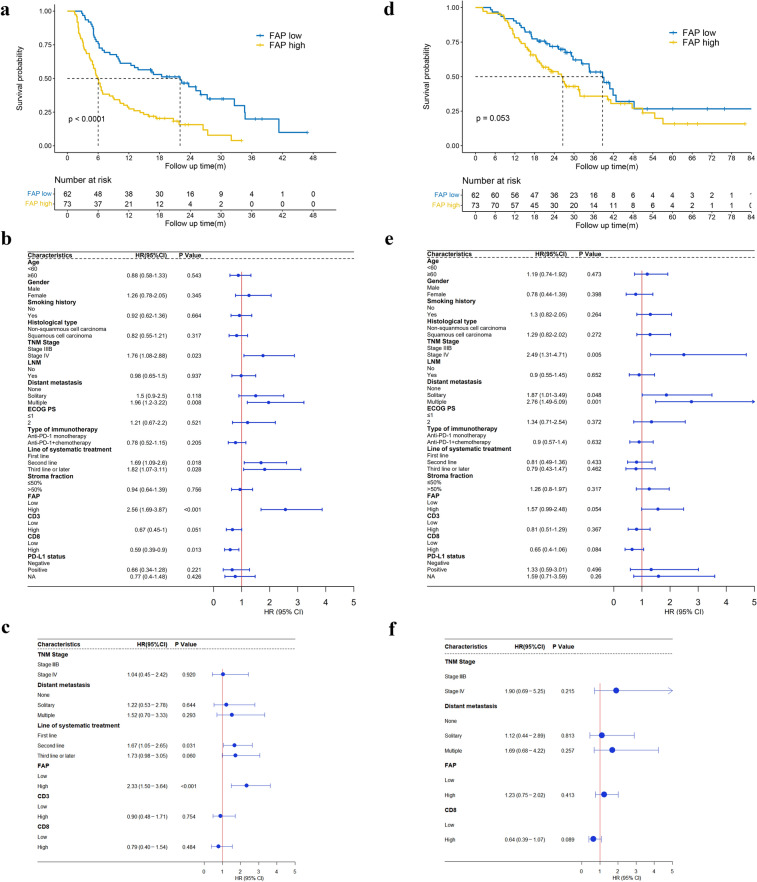
PFS and OS in the total study population. **a** Kaplan–Meier curves with patients stratified according to FAP expression (low *vs.* high) for PFS. **b** Forest plots of PFS, as determined by univariate Cox proportional hazards model. **c** Forest plots of PFS, as determined by multivariate Cox proportional hazards model, which were constructed using variables significantly associated with PFS by univariate Cox proportional hazards model (*p* < 0.1). **d** Kaplan‒Meier curves with patients stratified according to FAP expression (low *vs.* high) for OS. **e** Forest plots of OS, as determined by univariate Cox proportional hazards model. **f** Forest plots of OS as determined by multivariate Cox proportional hazards model, which were constructed using variables significantly associated with OS by univariate Cox proportional hazards model (*p* < 0.1)

Next, we confirmed that higher FAP expression was correlated with shortened PFS by Kaplan‒Meier analysis. The median PFS for patients with high expression of FAP was 6 months compared with 22 months for those with low expression of FAP, indicating that NSCLC patients with high FAP expression were more resistant to PD-1 blockade therapy (HR 2.56, 95% CI 1.69–3.87, *p* < 0.0001; Fig. 5a). Univariate analysis of the Cox proportional hazards model revealed that the factors significantly associated with PFS were TNM stage, distant metastasis, line of systematic therapy, FAP expression, and CD8 + T cell density, whereas age, gender, histology, ECOG PS, type of immunotherapy, stroma fraction, and CD3 + T cell density were not associated with the PFS of advanced NSCLC patients (Fig. 5b and Supplementary Fig. 2a–e).

To further evaluate the predictive role of FAP in PD-1 blockade therapy, clinicopathological parameters that were screened out in univariate analysis (*p* < 0.1) were included in multivariate survival analysis (Hang et al. 2021). Consequently, FAP expression (HR 2.33, 95% CI 1.50–3.64, *p* < 0.001) and line of systematic therapy (HR 1.67, 95% CI 1.05–2.65, *p* = 0.031) were independent prognostic factors for predicting PFS (Fig. 5c). Collectively, these analysis results demonstrated that for the NSCLC patients in an advanced stage, low expression of FAP and first-line immunotherapy might be factors predicting better efficacy of PD-1 blockade therapy.

Similarly, we assessed the predictive value of FAP in terms of OS in advanced NSCLC patients. The median OS for patients with low and high FAP expression was 38.7 months and 26.6 months, respectively, indicating that higher expression of FAP exhibited a correlation with shortened OS, although this correlation was not statistically significant (HR 1.57, 95% CI 0.99–2.48, *p* = 0.053; Fig. 5d). Univariate and multivariate analysis indicated that none of the variables were independent factors for predicting OS of NSCLC patients in advanced stages (Fig. 5e, f and Supplementary Fig. 3a–e).

Moreover, to investigate whether FAP was associated with clinical outcomes in the subpopulation of this cohort, a set of additional analyses were carried out. In the subgroups of squamous cell lung cancer and non-squamous cell lung cancer, PFS was negatively related to the expression of FAP, with HRs of 2.751 and 2.323, respectively (Supplementary Fig. 4a, c). In addition, a significant survival association was found for high FAP expression with lower OS in the subpopulation of squamous cell lung cancer patients (HR 2.19, 95% CI 1.11–4.32, *p* = 0.02, Supplementary Fig. 4d). Continued analysis was performed on subsets of NSCLC patients who received PD-1 blockade monotherapy, and an association of high FAP expression with shortened PFS was also observed (HR 4.28, 95% CI 2.22–8.28, *p* < 0.0001, Supplementary Fig. 4e). More strikingly, we found that the FAP expression was negatively associated with OS in the subpopulation of the PD-1 blockade monotherapy subgroup (HR 2.36, 95% CI 1.14–4.89, *p* = 0.017, Supplementary Fig. 4f), whereas this association was not observed in the combined treatment group with chemotherapy (Supplementary Fig. 4 g, h), suggesting that FAP might play a nonnegligible predictive and prognostic role in the cohort treated with PD-1 blockade monotherapy. Similar results were found in the subpopulation treated with first-line PD-1 blockade therapy (PFS: HR 4.11, 95% CI 1.97–8.58, *p* < 0.0001; OS: HR 2.32, 95% CI 1.07–5.03, *p* = 0.028, Supplementary Fig. 4i, j).

### The combination of FAP expression and CD8 + T cell density predicts the responsiveness to PD-1 blockade

T lymphocyte infiltration in tumors has been reported as a prerequisite for responsiveness to PD-1 blockade (Waldman et al. 2020; Zhang et al. 2019; Petitprez et al. 2020), We next confirmed that strong infiltration of CD8 + T cells was associated with a favorable response to PD-1 blockade (Fig. 5b and Supplementary Fig. 2e). Additionally, the median OS for patients with high CD8 + T cell infiltration in the tumor was 40.2 months compared with 29.2 months for those with low CD8 + T cell infiltration. However, the difference in OS was not statistically significant (*p* = 0.082, Supplementary Fig. 3e), with an unadjusted HR of 0.65 (95% CI 0.40–1.06).

**Fig. 6:**
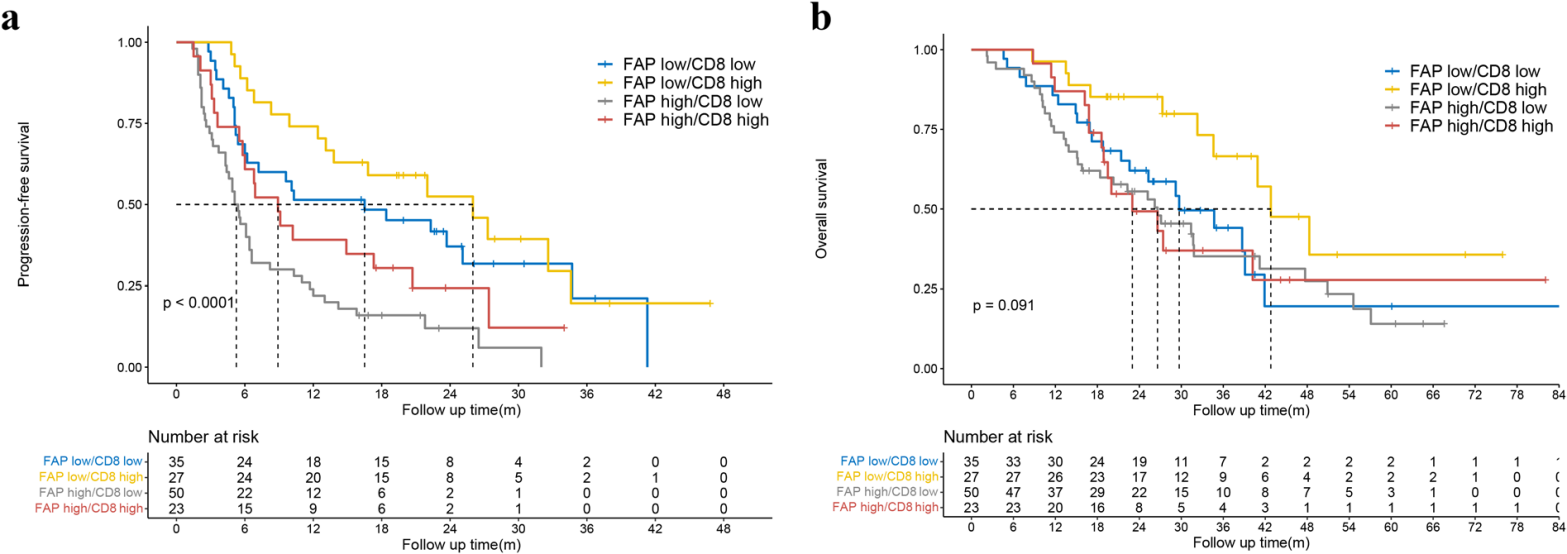
Kaplan‒Meier curves with patients stratified according to FAP expression and CD8 + T cell density for PFS (**a**) and OS (**b**)

As the above results indicated that the mere presence of CD8 + T cells was not entirely informative of whether advanced NSCLC patients were likely to respond to PD-1 blockade therapy, we next classified these patients into four groups based on the expression level of FAP and CD8 + T cell density: FAP low/CD8 low (*n* = 35), FAP low/CD8 high (*n* = 27), FAP high/CD8 low (*n* = 50) and FAP high/CD8 high (*n* = 23). The PFS of the FAP low/CD8 low, FAP low/CD8 high, FAP high/CD8 low and FAP high/CD8 high groups was 16.5, 26.0, 5.1 and 8.9 months, respectively. The PFS of the FAP low/CD8 high group was significantly longer than that of FAP high/CD8 low group (HR 0.26, 95% CI 0.14–0.48, *p* < 0.001), the FAP high/CD8 high group (HR 0.44, 95% CI 0.22–0.87, *p* = 0.015), and the FAP low/CD8 low group (HR 0.76, 95% CI 0.40–1.43, *p* = 0.393) (Fig. 6a). Similar trends were also observed in OS (Fig. 6b). Taken together, these results provided evidence that simultaneous evaluation of FAP and CD8 + T cells had better directive significance for predicting the prognosis of NSCLC patients who received PD-1 blockade therapy.

## Discussion

Engagement of oncogenic factors in the tumoral stroma has been proposed as a critical mechanism that remodels immunosuppressive features of the TME and leads to resistance to ICB. Therefore, more focus on the tumor stroma and a priori identification of responders to ICB is of utmost importance to improve clinical outcomes. In this study, we first identified the clinical significance of a CAF biomarker, FAP expression in the stroma, with respect to immunotherapy outcomes in advanced NSCLC patients. Analysis of patient prognosis demonstrated that high FAP expression, together with later-line immunotherapy, was significantly associated with a shortened PFS. In addition, the abundance of FAP-positive cells was negatively associated with the density of CD8 + T cells. Our study highlights the potential of stromal FAP expression as a companion biomarker for immunotherapy and reveals that targeting FAP is a promising strategy for increasing T cell infiltration and overcoming resistance to PD-1 blockade therapy in NSCLC.

In this study, we examined the association between FAP and clinicopathologic parameters in our advanced NSCLC cohort and found that FAP was correlated with distant metastasis, which proved the tumor-promoting function of FAP in the invasion and metastasis of cancer (Woo et al. 2022). Then, we found that high expression of FAP was correlated with a reduced response to PD-1 blockade, with a decreased ORR and DCR as well as shortened PFS. Although the evidence in relation to FAP and OS was not statistically significant, the data showed a trend that high expression of FAP resulted in shortened OS in advanced NSCLC. Nevertheless, some results in this study were not consistent with previous studies, depending on the IHC scoring system for evaluating the expression of FAP. In previous studies, the expression of FAP referred to the FAP intensity or the fraction of FAP-positive stromal cells (Henry et al. 2007; Herrera et al. 2020), whereas in this study, we integrated FAP intensity and the fraction of FAP-positive stromal cells into a total IHC score. Moreover, we enrolled advanced-stage NSCLC patients with distant metastasis, accounting for 69.9% of our cohort, which was different from studies that only included resectable NSCLC patients in early stages (Kilvaer et al. 2018). Thus, additional large-sample multicenter studies exploring the role of FAP in the prediction of immunotherapeutic outcomes and the prognosis of NSCLC are warranted.

ICBs reinvigorate an efficacious antitumor immune response by taking advantage of TILs in the TME. Thus, the practical efficacy of ICBs largely depends on the abundance of TILs. However, insufficient infiltration of T cells is a hallmark of the immunosuppressive nature of the TME and represents a major form of primary resistance to PD-1 blockade therapy (Joyce and Fearon 2015). Experimental evidence has indicated that FAP-expressing stromal cells contact T cells at the outer edge of the tumor and restrict the proliferation and intratumoral recruitment of effector T cells in various cancers (Ogawa et al. 2021; Feig et al. 2013; Cremasco et al. 2018). In addition, FAP + CAFs impaired the killing activity of CD8 + T cells by affecting the NF-κB signaling pathway and upregulated the expression of immune checkpoint receptors (Érsek et al. 2020). Consistent with the immunosuppressive function of FAP mentioned above, we found that FAP expression was inversely correlated with CD8 + T cell density in our cohort as well as in the TIMER database. However, several previous studies failed to detect associations between FAP and CD8 + T cell infiltration (Moreno-Ruiz et al. 2021; Wong et al. 2019), indicating that the role of FAP is highly heterogeneous. Additionally, specific CD8 + TILs subgroups rather than the total CD8 + T cells might characterize the TME and predict therapeutic effects. For example, high infiltration level of CD8 + PD-L1 + TILs indicated a strongly immunosuppressive TME but was associated with better response to PD-1 blockade (Zhang et al. 2021). Thus, future studies that focus on the tumor immune microenvironment merit further investigations and will likely shed light on mechanisms underlying fibroblast-immune crosstalk.

To the best of our knowledge, this study is the first to reveal that FAP is a potential biomarker for predicting the response to PD-1 blockade therapy in advanced NSCLC patients. However, the present study has several limitations. The major limitation is the nonrandomized, retrospective, heterogeneous nature of the cohort, involving multiple types of immunotherapies (monotherapy or combined with chemotherapy) and patients treated in second or later lines with PD-1 blockade. However, we considered these parameters in the Cox proportional hazards model and found that FAP was an independent factor for predicting PFS. Another limitation is that we evaluated the density of infiltrating T lymphocytes but did not further investigate other immune cell types in the TME, due to the limited availability of tumor tissues from advanced NSCLC patients. Instead, we utilized the TIMER database and found that FAP was positively associated with the abundance of immunosuppressive cells. Notwithstanding, more direct evidence is needed to elucidate the regulatory mechanisms of FAP on immune-related features. Overall, the promising results encouraged us to carry out larger, randomized controlled trials in the future and focus on the immunogenic characteristics of FAP-positive CAFs and the mechanisms of fibroblast-immune crosstalk with immunotherapeutic potential in NSCLC.

Currently, PET imaging, a noninvasive imaging technique, has been proven to be a promising tool to quantitatively monitor cell dynamics in the TME. Quinolone-based FAP inhibitors (FAPIs) have been successfully applied to PET imaging in various cancers, providing advantages over conventional ^18^F-FDG PET/CT in several tumor entities for the initial staging and detection of tumor recurrence and metastases (Pang et al. 2021). In a recent study, researchers used ^68^ Ga-FAPI-04 PET/CT as a noninvasive biomarker to predict outcomes of ICB therapy in gastric cancer, demonstrating that patients with higher FAPI-uptake failed to benefit from ICB therapy (Rong et al. 2021). Moreover, the ^68^ Ga-FAPI-46 PET biodistribution was strongly similar to the FAP expression pattern detected by IHC in tumor tissues (Mona et al. 2021). Although the correlation between FAPI PET/CT and immunotherapy response was not explored in our study, we shed light on the potential clinical utility of FAPI PET/CT for the prediction of an immunotherapeutic response in NSCLC patients, facilitating the development of multidisciplinary strategies in immunotherapy.

## Conclusion

This study analyzed the clinical significance of a canonical CAF marker, FAP, in pretreatment tumor specimens to determine its association with T cell infiltration and immunotherapy outcomes in advanced NSCLC. Our results demonstrate that high expression of FAP is associated with lower proportions of CD8 + T cells, which dampen antitumor immunity and thus facilitate immune escape. Moreover, FAP is an indicative biomarker for immunotherapeutic resistance and shows a negative correlation with PFS independent of baseline variables in multivariable analyses. We highlight the potential application of FAP as a companion biomarker in immuno-oncology, as FAP might guide stroma-based stratification of patients to predict the immunotherapeutic response and outcomes of PD-1 blockade therapy. Finally, our findings provide evidence that targeting FAP is a potential strategy for alleviating the immunosuppressive TME and overcoming resistance to PD-1 blockade in NSCLC.

## Supplementary Information

Below is the link to the electronic supplementary material.

Supplementary file1 (TIF 22150 KB) Supplementary Fig. 1. Representative IHC images of tumors showing positive and negative staining of PD-L1. Scale bar = 50 µm

Supplementary file2 (TIF 33840 KB) Supplementary Fig. 2. Kaplan–Meier curves for PFS applied to different clinical groups: TNM stage (a), distant metastasis (b), line of systematic therapy (c), CD3+ T cell density (d) and CD8+ T cell density (e)

Supplementary file3 (TIF 33840 KB) Supplementary Fig. 3. Kaplan‒Meier curves for OS applied to different clinical groups: TNM stage (a), distant metastasis (b), line of systematic therapy (c), CD3+ T cell density (d) and CD8+ T cell density (e)

Supplementary file4 (TIF 110591 KB) Supplementary Fig. 4. Subgroup analysis of advanced NSCLC patients. Kaplan‒Meier curves with patients stratified according to FAP expression (low vs. high) in non-squamous cell lung cancer for PFS (a) and OS (b), in squamous cell lung cancer for PFS (c) and OS (d), in the PD-1 blockade monotherapy group for PFS (e) and OS (f), in the PD-1 blockade combined with platinum-based chemotherapy group for PFS (g) and OS (h), and in the first-line therapy group for PFS (i) and OS (j)

## Data Availability

The datasets generated and/or analyzed during the present study are available from the corresponding author on reasonable request.

## Abbreviations

NSCLC: Non-small cell lung cancer
PD-1: Programmed cell death-1
PD-L1: Programmed cell death ligand-1
TME: Tumor microenvironment
CAFs: Cancer-associated fibroblasts
FAP: Fibroblast activation protein
TILs: Tumor infiltrating lymphocytes
LUAD: Lung adenocarcinoma
LUSC: Lung squamous cell carcinoma
IHC: Immunohistochemistry
CR: Complete response
PR: Partial response
SD: Stable disease
PD: Progressive disease
ORR: Objective response rate
DCR: Disease control rate
PFS: Progression-free survival
OS: Overall survival
FAPI: Quinolone-based FAP inhibitor

## References

[CR1] Bagaev A, Kotlov N, Nomie K, Svekolkin V, Gafurov A, Isaeva O et al (2021) Conserved pan-cancer microenvironment subtypes predict response to immunotherapy. Cancer Cell 39:845–65.e7. 10.1016/j.ccell.2021.04.014 34019806 10.1016/j.ccell.2021.04.014

[CR2] Bouchard G, Garcia-Marques FJ, Karacosta LG, Zhang W, Bermudez A, Riley NM et al (2022) Multiomics analysis of spatially distinct stromal cells reveals tumor-induced O-glycosylation of the CDK4-pRB axis in fibroblasts at the invasive tumor edge. Cancer Res 82:648–664. 10.1158/0008-5472.Can-21-1705 34853070 10.1158/0008-5472.CAN-21-1705 PMC9075699

[CR3] Carbone DP, Reck M, Paz-Ares L, Creelan B, Horn L, Steins M et al (2017) First-line nivolumab in stage IV or recurrent non-small-cell lung cancer. N Engl J Med 376:2415–2426. 10.1056/NEJMoa1613493 28636851 10.1056/NEJMoa1613493 PMC6487310

[CR4] Chen L, Qiu X, Wang X, He J (2017) FAP positive fibroblasts induce immune checkpoint blockade resistance in colorectal cancer via promoting immunosuppression. Biochem Biophys Res Commun 487:8–14. 10.1016/j.bbrc.2017.03.039 28302482 10.1016/j.bbrc.2017.03.039

[CR5] Costa A, Kieffer Y, Scholer-Dahirel A, Pelon F, Bourachot B, Cardon M et al (2018) Fibroblast heterogeneity and immunosuppressive environment in human breast cancer. Cancer Cell 33:463–79.e10. 10.1016/j.ccell.2018.01.011 29455927 10.1016/j.ccell.2018.01.011

[CR6] Cremasco V, Astarita JL, Grauel AL, Keerthivasan S, MacIsaac K, Woodruff MC et al (2018) FAP delineates heterogeneous and functionally divergent stromal cells in immune-excluded breast tumors. Cancer Immunol Res 6:1472–1485. 10.1158/2326-6066.Cir-18-0098 30266714 10.1158/2326-6066.CIR-18-0098 PMC6597261

[CR7] Dominguez CX, Müller S, Keerthivasan S, Koeppen H, Hung J, Gierke S et al (2020) Single-cell RNA sequencing reveals stromal evolution into LRRC15(+) myofibroblasts as a determinant of patient response to cancer immunotherapy. Cancer Discov 10:232–253. 10.1158/2159-8290.Cd-19-0644 31699795 10.1158/2159-8290.CD-19-0644

[CR8] Dong ZY, Zhong WZ, Zhang XC, Su J, Xie Z, Liu SY et al (2017) Potential predictive value of TP53 and KRAS mutation status for response to PD-1 blockade immunotherapy in lung adenocarcinoma. Clin Cancer Res 23:3012–3024. 10.1158/1078-0432.Ccr-16-2554 28039262 10.1158/1078-0432.CCR-16-2554

[CR9] Eisenhauer EA, Therasse P, Bogaerts J, Schwartz LH, Sargent D, Ford R et al (2009) New response evaluation criteria in solid tumours: revised RECIST guideline (version 1.1). Eur J Cancer 45:228–247. 10.1016/j.ejca.2008.10.026 19097774 10.1016/j.ejca.2008.10.026

[CR10] Érsek B, Silló P, Cakir U, Molnár V, Bencsik A, Mayer B et al (2020) Melanoma-associated fibroblasts impair CD8+ T cell function and modify expression of immune checkpoint regulators via increased arginase activity. Cell Mol Life Sci. 10.1007/s00018-020-03517-8 32328671 10.1007/s00018-020-03517-8 PMC7581550

[CR11] Feig C, Jones JO, Kraman M, Wells RJ, Deonarine A, Chan DS et al (2013) Targeting CXCL12 from FAP-expressing carcinoma-associated fibroblasts synergizes with anti-PD-L1 immunotherapy in pancreatic cancer. Proc Natl Acad Sci U S A 110:20212–20217. 10.1073/pnas.1320318110 24277834 10.1073/pnas.1320318110 PMC3864274

[CR12] Fitzgerald AA, Weiner LM (2020) The role of fibroblast activation protein in health and malignancy. Cancer Metastasis Rev 39:783–803. 10.1007/s10555-020-09909-3 32601975 10.1007/s10555-020-09909-3 PMC7487063

[CR13] Galbo PM Jr, Zang X, Zheng D (2021) Molecular features of cancer-associated fibroblast subtypes and their implication on cancer pathogenesis prognosis, and immunotherapy resistance. Clin Cancer Res 27:2636–2647. 10.1158/1078-0432.Ccr-20-4226 33622705 10.1158/1078-0432.CCR-20-4226 PMC8102353

[CR14] Galon J, Costes A, Sanchez-Cabo F, Kirilovsky A, Mlecnik B, Lagorce-Pagès C et al (2006) Type, density, and location of immune cells within human colorectal tumors predict clinical outcome. Science 313:1960–1964. 10.1126/science.1129139 17008531 10.1126/science.1129139

[CR15] Gandhi L, Rodríguez-Abreu D, Gadgeel S, Esteban E, Felip E, De Angelis F et al (2018) Pembrolizumab plus chemotherapy in metastatic non-small-cell lung cancer. N Engl J Med 378:2078–2092. 10.1056/NEJMoa1801005 29658856 10.1056/NEJMoa1801005

[CR16] Garon EB, Rizvi NA, Hui R, Leighl N, Balmanoukian AS, Eder JP et al (2015) Pembrolizumab for the treatment of non-small-cell lung cancer. N Engl J Med 372:2018–2028. 10.1056/NEJMoa1501824 25891174 10.1056/NEJMoa1501824

[CR17] Hang J, Xu K, Yin R, Shao Y, Liu M, Shi H et al (2021) Role of CT texture features for predicting outcome of pancreatic cancer patients with liver metastases. J Cancer 12:2351–2358. 10.7150/jca.49569 33758611 10.7150/jca.49569 PMC7974874

[CR18] Henry LR, Lee HO, Lee JS, Klein-Szanto A, Watts P, Ross EA et al (2007) Clinical implications of fibroblast activation protein in patients with colon cancer. Clin Cancer Res 13:1736–1741. 10.1158/1078-0432.Ccr-06-1746 17363526 10.1158/1078-0432.CCR-06-1746

[CR19] Herbst RS, Baas P, Kim DW, Felip E, Pérez-Gracia JL, Han JY et al (2016) Pembrolizumab versus docetaxel for previously treated, PD-L1-positive, advanced non-small-cell lung cancer (KEYNOTE-010): a randomised controlled trial. Lancet 387:1540–1550. 10.1016/s0140-6736(15)01281-7 26712084 10.1016/S0140-6736(15)01281-7

[CR20] Herrera M, Mezheyeuski A, Villabona L, Corvigno S, Strell C, Klein C et al (2020) Prognostic interactions between FAP+ fibroblasts and CD8a+ T cells in colon cancer. Cancers (basel). 10.3390/cancers12113238 33153037 10.3390/cancers12113238 PMC7693786

[CR21] Hirsch L, Zitvogel L, Eggermont A, Marabelle A (2019) PD-Loma: a cancer entity with a shared sensitivity to the PD-1/PD-L1 pathway blockade. Br J Cancer 120:3–5. 10.1038/s41416-018-0294-4 30413824 10.1038/s41416-018-0294-4 PMC6325162

[CR22] Hu H, Piotrowska Z, Hare PJ, Chen H, Mulvey HE, Mayfield A et al (2021) Three subtypes of lung cancer fibroblasts define distinct therapeutic paradigms. Cancer Cell 39:1531–47.e10. 10.1016/j.ccell.2021.09.003 34624218 10.1016/j.ccell.2021.09.003 PMC8578451

[CR23] Ilie M, Long-Mira E, Bence C, Butori C, Lassalle S, Bouhlel L et al (2016) Comparative study of the PD-L1 status between surgically resected specimens and matched biopsies of NSCLC patients reveal major discordances: a potential issue for anti-PD-L1 therapeutic strategies. Ann Oncol 27:147–153. 10.1093/annonc/mdv489 26483045 10.1093/annonc/mdv489

[CR24] Jiang P, Gu S, Pan D, Fu J, Sahu A, Hu X et al (2018) Signatures of T cell dysfunction and exclusion predict cancer immunotherapy response. Nat Med 24:1550–1558. 10.1038/s41591-018-0136-1 30127393 10.1038/s41591-018-0136-1 PMC6487502

[CR25] Joyce JA, Fearon DT (2015) T cell exclusion, immune privilege, and the tumor microenvironment. Science 348:74–80. 10.1126/science.aaa6204 25838376 10.1126/science.aaa6204

[CR26] Kieffer Y, Hocine HR, Gentric G, Pelon F, Bernard C, Bourachot B et al (2020) Single-cell analysis reveals fibroblast clusters linked to immunotherapy resistance in cancer. Cancer Discov 10:1330–1351. 10.1158/2159-8290.Cd-19-1384 32434947 10.1158/2159-8290.CD-19-1384

[CR27] Kilvaer TK, Rakaee M, Hellevik T, Østman A, Strell C, Bremnes RM et al (2018) Tissue analyses reveal a potential immune-adjuvant function of FAP-1 positive fibroblasts in non-small cell lung cancer. PLoS ONE 13:e0192157. 10.1371/journal.pone.0192157 29415055 10.1371/journal.pone.0192157 PMC5802915

[CR28] Kraman M, Bambrough PJ, Arnold JN, Roberts EW, Magiera L, Jones JO et al (2010) Suppression of antitumor immunity by stromal cells expressing fibroblast activation protein-alpha. Science 330:827–830. 10.1126/science.1195300 21051638 10.1126/science.1195300

[CR29] Li T, Fu J, Zeng Z, Cohen D, Li J, Chen Q et al (2020) TIMER2.0 for analysis of tumor-infiltrating immune cells. Nucleic Acids Res 48:W509–W514. 10.1093/nar/gkaa407 32442275 10.1093/nar/gkaa407 PMC7319575

[CR30] Mansfield AS, Aubry MC, Moser JC, Harrington SM, Dronca RS, Park SS et al (2016) Temporal and spatial discordance of programmed cell death-ligand 1 expression and lymphocyte tumor infiltration between paired primary lesions and brain metastases in lung cancer. Ann Oncol 27:1953–1958. 10.1093/annonc/mdw289 27502709 10.1093/annonc/mdw289 PMC5035793

[CR31] Mona CE, Benz MR, Hikmat F, Grogan TR, Lückerath K, Razmaria A et al (2021) Correlation of ^68^Ga-FAPi-46 PET biodistribution with FAP expression by immunohistochemistry in patients with solid cancers: a prospective translational exploratory study. J Nucl Med. 10.2967/jnumed.121.262426 34740953 10.2967/jnumed.121.262426 PMC9258565

[CR32] Moreno-Ruiz P, Corvigno S, Te Grootenhuis NC, La Fleur L, Backman M, Strell C et al (2021) Stromal FAP is an independent poor prognosis marker in non-small cell lung adenocarcinoma and associated with p53 mutation. Lung Cancer 155:10–19. 10.1016/j.lungcan.2021.02.028 33706022 10.1016/j.lungcan.2021.02.028

[CR33] Ogawa Y, Masugi Y, Abe T, Yamazaki K, Ueno A, Fujii-Nishimura Y et al (2021) Three distinct stroma types in human pancreatic cancer identified by image analysis of fibroblast subpopulations and collagen. Clin Cancer Res 27:107–119. 10.1158/1078-0432.Ccr-20-2298 33046515 10.1158/1078-0432.CCR-20-2298

[CR34] Pang Y, Zhao L, Luo Z, Hao B, Wu H, Lin Q et al (2021) Comparison of ^68^ Ga-FAPI and ^18^ F-FDG uptake in gastric, duodenal, and colorectal cancers. Radiology 298:393–402. 10.1148/radiol.2020203275 33258746 10.1148/radiol.2020203275

[CR35] Petitprez F, Meylan M, de Reyniès A, Sautès-Fridman C, Fridman WH (2020) The tumor microenvironment in the response to immune checkpoint blockade therapies. Front Immunol 11:784. 10.3389/fimmu.2020.00784 32457745 10.3389/fimmu.2020.00784 PMC7221158

[CR36] Powles T, Kockx M, Rodriguez-Vida A, Duran I, Crabb SJ, Van Der Heijden MS et al (2019) Clinical efficacy and biomarker analysis of neoadjuvant atezolizumab in operable urothelial carcinoma in the ABACUS trial. Nat Med 25:1706–1714. 10.1038/s41591-019-0628-7 31686036 10.1038/s41591-019-0628-7

[CR37] Reck M, Remon J, Hellmann MD (2022) First-line immunotherapy for non-small-cell lung cancer. J Clin Oncol 40:586–597. 10.1200/jco.21.01497 34985920 10.1200/JCO.21.01497

[CR38] Renga G, Nunzi E, Pariano M, Puccetti M, Bellet MM, Pieraccini G et al (2022) Optimizing therapeutic outcomes of immune checkpoint blockade by a microbial tryptophan metabolite. J Immunother Cancer. 10.1136/jitc-2021-003725 35236743 10.1136/jitc-2021-003725 PMC8896050

[CR39] Rong X, Lv J, Liu Y, Wang Z, Zeng D, Li Y et al (2021) PET/CT imaging of activated cancer-associated fibroblasts predict response to PD-1 blockade in gastric cancer patients. Front Oncol 11:802257. 10.3389/fonc.2021.802257 35155199 10.3389/fonc.2021.802257 PMC8825781

[CR40] Sideras K, Biermann K, Yap K, Mancham S, Boor PPC, Hansen BE et al (2017) Tumor cell expression of immune inhibitory molecules and tumor-infiltrating lymphocyte count predict cancer-specific survival in pancreatic and ampullary cancer. Int J Cancer 141:572–582. 10.1002/ijc.30760 28470686 10.1002/ijc.30760

[CR41] Subramanian A, Tamayo P, Mootha VK, Mukherjee S, Ebert BL, Gillette MA et al (2005) Gene set enrichment analysis: a knowledge-based approach for interpreting genome-wide expression profiles. Proc Natl Acad Sci U S A 102:15545–15550. 10.1073/pnas.0506580102 16199517 10.1073/pnas.0506580102 PMC1239896

[CR42] Sun D, Wang J, Han Y, Dong X, Ge J, Zheng R et al (2021) TISCH: a comprehensive web resource enabling interactive single-cell transcriptome visualization of tumor microenvironment. Nucleic Acids Res 49:D1420–D1430. 10.1093/nar/gkaa1020 33179754 10.1093/nar/gkaa1020 PMC7778907

[CR43] Waldman AD, Fritz JM, Lenardo MJ (2020) A guide to cancer immunotherapy: from T cell basic science to clinical practice. Nat Rev Immunol 20:651–668. 10.1038/s41577-020-0306-5 32433532 10.1038/s41577-020-0306-5 PMC7238960

[CR44] Wang Y, Liang Y, Xu H, Zhang X, Mao T, Cui J et al (2021) Single-cell analysis of pancreatic ductal adenocarcinoma identifies a novel fibroblast subtype associated with poor prognosis but better immunotherapy response. Cell Discov 7:36. 10.1038/s41421-021-00271-4 34035226 10.1038/s41421-021-00271-4 PMC8149399

[CR45] Wankhede D (2021) Evaluation of eighth AJCC TNM sage for lung cancer NSCLC: a meta-analysis. Ann Surg Oncol 28:142–147. 10.1245/s10434-020-09151-9 32951092 10.1245/s10434-020-09151-9

[CR46] Wen X, He X, Jiao F, Wang C, Sun Y, Ren X et al (2017) Fibroblast activation protein-α-positive fibroblasts promote gastric cancer progression and resistance to immune checkpoint blockade. Oncol Res 25:629–640. 10.3727/096504016x14768383625385 27983931 10.3727/096504016X14768383625385 PMC7841289

[CR47] Wong PF, Wei W, Gupta S, Smithy JW, Zelterman D, Kluger HM et al (2019) Multiplex quantitative analysis of cancer-associated fibroblasts and immunotherapy outcome in metastatic melanoma. J Immunother Cancer 7:194. 10.1186/s40425-019-0675-0 31337426 10.1186/s40425-019-0675-0 PMC6651990

[CR48] Woo HY, Rhee H, Yoo JE, Kim SH, Choi GH, Kim DY et al (2022) Lung and lymph node metastases from hepatocellular carcinoma: comparison of pathological aspects. Liver Int 42:199–209. 10.1111/liv.15051 34490997 10.1111/liv.15051

[CR49] Yang X, Lin Y, Shi Y, Li B, Liu W, Yin W et al (2016) FAP promotes immunosuppression by cancer-associated fibroblasts in the tumor microenvironment via STAT3-CCL2 signaling. Cancer Res 76:4124–4135. 10.1158/0008-5472.Can-15-2973 27216177 10.1158/0008-5472.CAN-15-2973

[CR50] Yoshihara K, Shahmoradgoli M, Martínez E, Vegesna R, Kim H, Torres-Garcia W et al (2013) Inferring tumor purity and stromal and immune cell admixture from expression data. Nat Commun 4:2612. 10.1038/ncomms3612 24113773 10.1038/ncomms3612 PMC3826632

[CR51] Zhang J, Endres S, Kobold S (2019) Enhancing tumor T cell infiltration to enable cancer immunotherapy. Immunotherapy 11:201–213. 10.2217/imt-2018-0111 30730277 10.2217/imt-2018-0111

[CR52] Zhang L, Chen Y, Wang H, Xu Z, Wang Y, Li S et al (2021) Massive PD-L1 and CD8 double positive TILs characterize an immunosuppressive microenvironment with high mutational burden in lung cancer. J Immunother Cancer. 10.1136/jitc-2021-002356 34140315 10.1136/jitc-2021-002356 PMC8212410

