# Supplementary figures and images for: Fibroblast activation protein in the tumor microenvironment predicts outcomes of PD-1 blockade therapy in advanced non-small cell lung cancer
Source: J Cancer Res Clin Oncol. 2022 Aug 11;149(7):3469–83. doi: 10.1007/s00432-022-04250-4 (PMC10314861; doi:10.1007/s00432-022-04250-4)

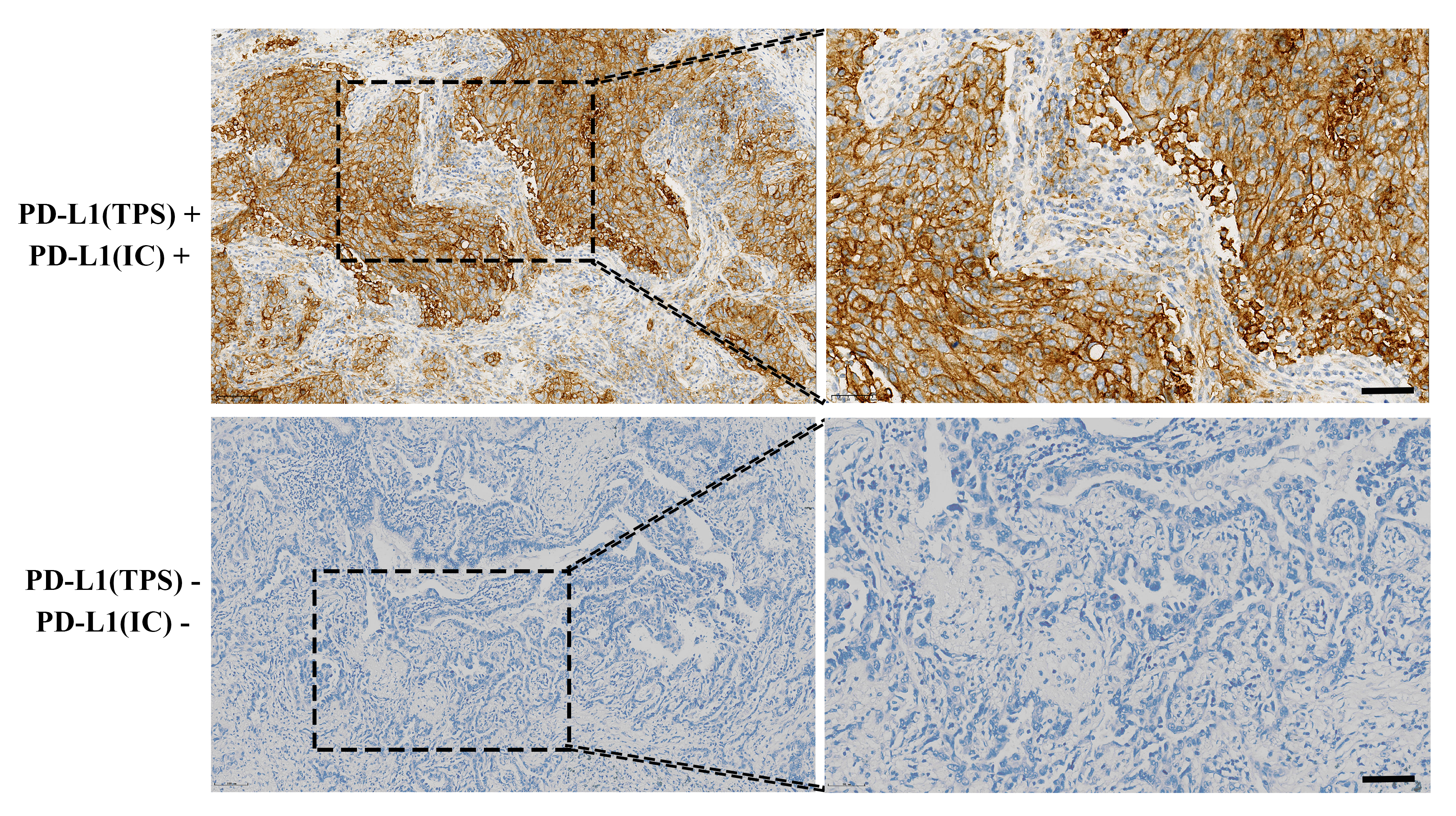

Supplement: Supplementary file 1 — Supplementary file1 (TIF 22150 KB) Supplementary Fig. 1. Representative IHC images of tumors showing positive and negative staining of PD-L1. Scale bar = 50 µm [file 432_2022_4250_MOESM1_ESM.tif]

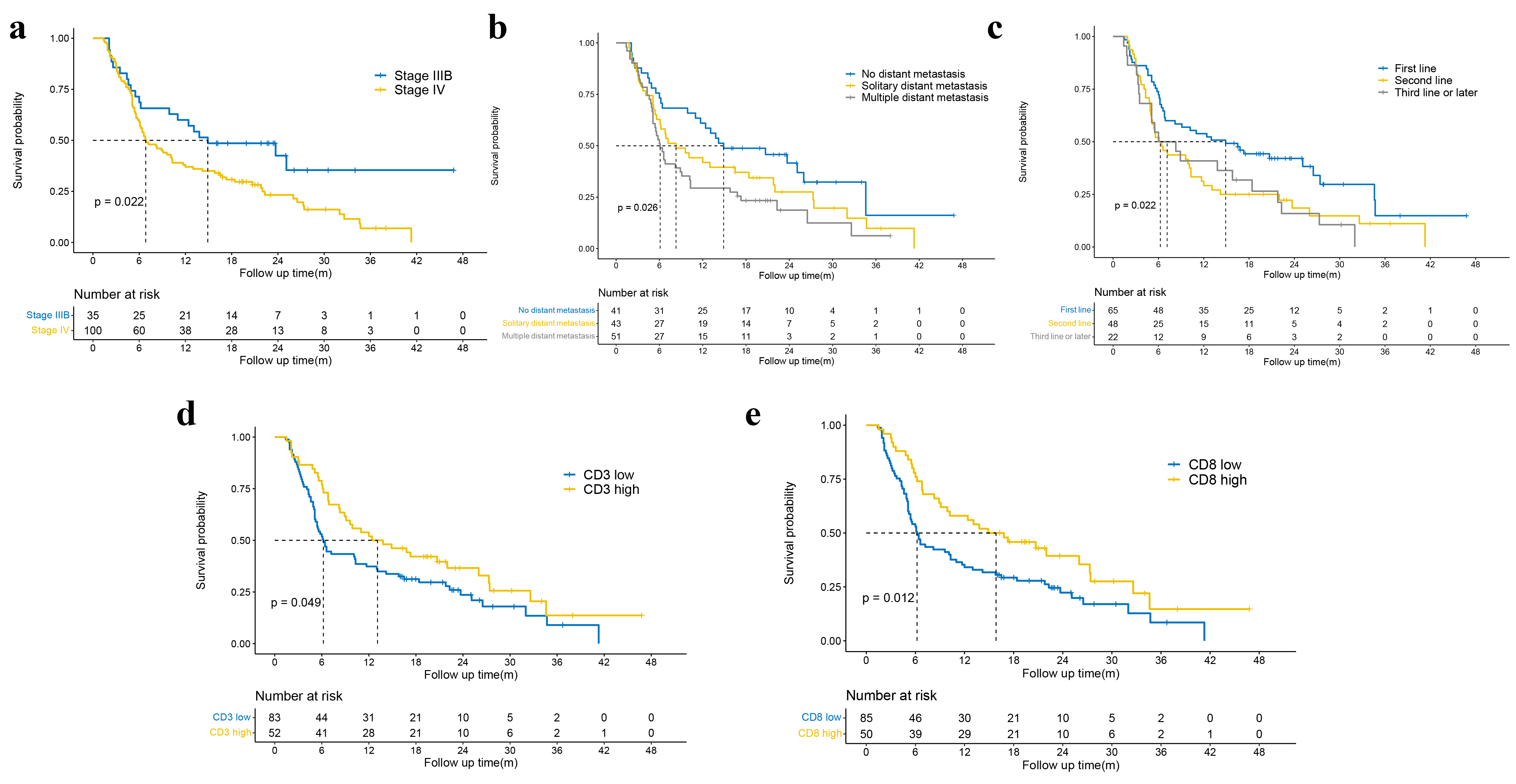

Supplement: Supplementary file 2 — Supplementary file2 (TIF 33840 KB) Supplementary Fig. 2. Kaplan–Meier curves for PFS applied to different clinical groups: TNM stage (a), distant metastasis (b), line of systematic therapy (c), CD3+ T cell density (d) and CD8+ T cell density (e) [file 432_2022_4250_MOESM2_ESM.tif]

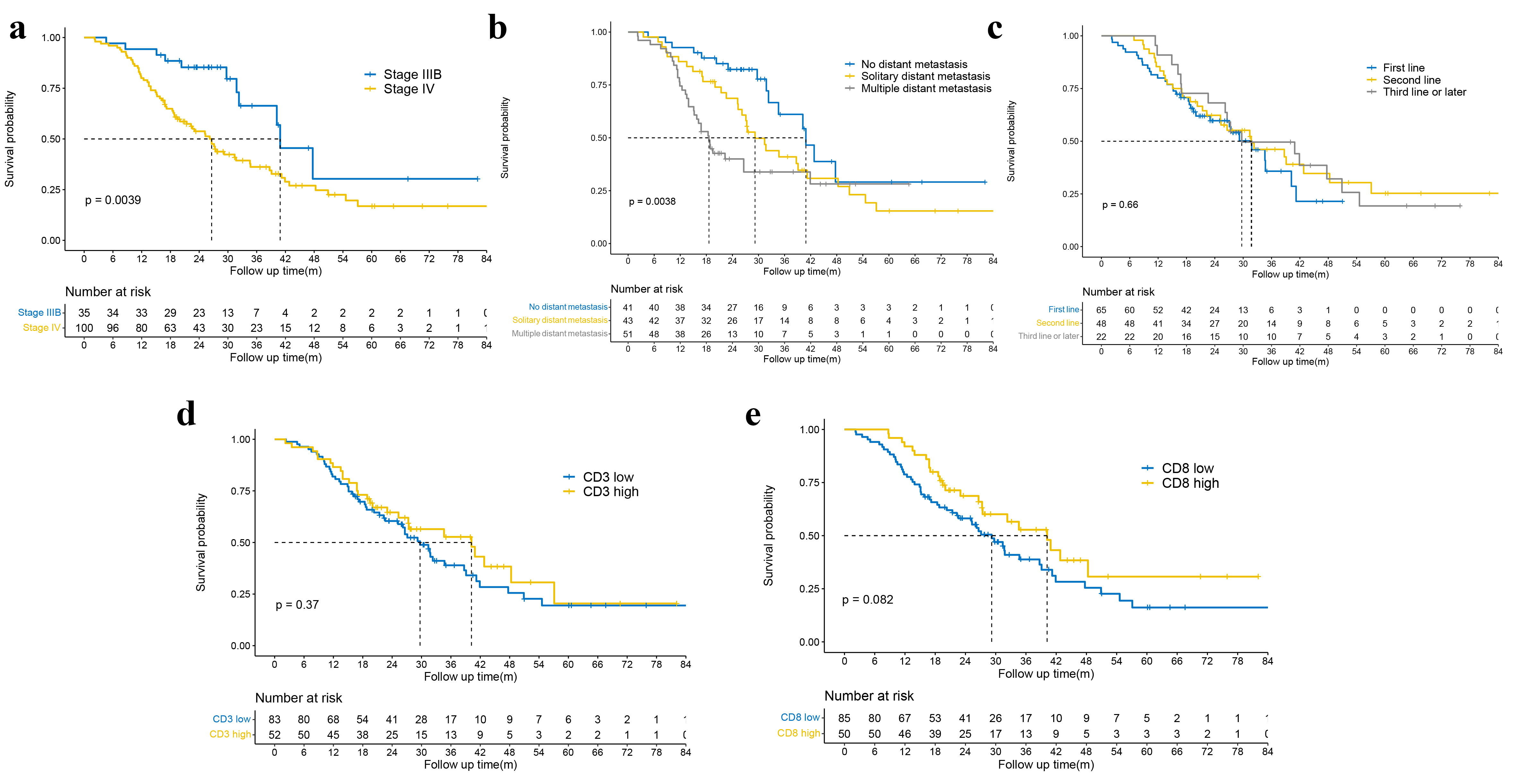

Supplement: Supplementary file 3 — Supplementary file3 (TIF 33840 KB) Supplementary Fig. 3. Kaplan‒Meier curves for OS applied to different clinical groups: TNM stage (a), distant metastasis (b), line of systematic therapy (c), CD3+ T cell density (d) and CD8+ T cell density (e) [file 432_2022_4250_MOESM3_ESM.tif]
